# Spatial Association between Gastric Cancer Mortality and Goiter in Sardinia

**DOI:** 10.31557/APJCP.2021.22.1.105

**Published:** 2021-01

**Authors:** Giovanni Mario Pes, Giuseppe Fanciulli, Alessandro Palmerio Delitala, Andrea Fausto Piana, Maria Pina Dore

**Affiliations:** 1 *Dipartimento di Scienze Mediche, Chirurgiche e Sperimentali, University of Sassari, Sassari 07100, Italy. *; 2 *Baylor College of Medicine, 77030 Houston, Texas, USA. *

**Keywords:** Gastric carcinoma, Helicobacter pylori, goiter, diet, stature, sheep, rearing

## Abstract

**Background:**

Gastric cancer (GC) is the third leading cause of cancer mortality worldwide. The incidence of GC varies between countries according to exposure to different risk factors. Hypothyroidism has been suggested as a potential GC risk factor. In Sardinia, Italy, the prevalence of endemic goiter is high and GC mortality is unevenly distributed. This ecological study aimed to investigate GC mortality and its relationship with hypothyroidism, adjusting for potential confounders.

**Methods:**

The spatial association between GC mortality and goiter (a proxy of hypothyroidism), diet, stature and pastoralism (a proxy of *Helicobacter pylori* infection), available at the aggregated level, was modelled in the island’s 377 municipalities, separately by sex, using geographically weighted regression (GWR).

**Results:**

The GC standardized mortality ratio ranged from 0.0 to 10.4 across municipalities. A hotspot of GC mortality was detected in the central mountainous area of Sardinia among males, positively associated with goiter (GWR estimate 0.213 ± 0.122), and the practice of sheep‒rearing (GWR estimate 0.127 ± 0.080), whereas a negative association with the diet score (GWR estimate 0.032 ± 0.034), and null for stature were found. No significant associations were found in females.

**Conclusion:**

Within the limitations of ecological studies goiter prevalence was an independent predictor of GC mortality in males.

## Introduction

Gastric cancer (GC) is the third leading cause of death among cancers and remains one of the most frequent malignancies diagnosed worldwide with 1,033,701 new cases (5.7% of all cancers) reported in 2018 (http://gco.iarc.fr/today/fact-sheets-cancers). Although a dramatic decline in GC mortality has been reported (i.e. ‒17.1%) in the decade 2007–2017 (Roth et al., 2018) the absolute number of GC cases diagnosed annually is increasing, with a trend shift toward younger generations (Correa, 2011). The reasons for this rise in GC incidence in young subjects are still incompletely understood and suggest the existence of additional risk factors for this malignancy behind the established ones. The incidence of GC varies across countries, and even within the same geographic region, depending in part upon exposure to various risk factors. The most affected populations are those of East Asia, Eastern Europe and South America, while GC has become infrequent in Western Europe, North America and most of Africa (Forman and Burley, 2006). In Italy, the incidence rate is about 10 cases per 100,000 inhabitants per year (https://www.aiom.it/wp-content/uploads/2018/10/2018_NumeriCancro-operatori.pdf). The Mediterranean island of Sardinia shows an epidemiological peculiarity: in 1999 Sardinia was the Italian region with the lowest standardized mortality ratio (SMR) for GC (0.97 against a national average of 1.72) (Atlante della Mortalità Sarda 1981‒1988. ISTAT, Roma, Italy, 2005). However, the distribution within the island is very heterogeneous, with the highest SMR in the central‒eastern area of the island known as “*Ogliastra*”, where the ratio exceeded twice the national average. Such marked difference suggests the existence of one or more risk factors for GC with a similar geographic distribution.

Although *Helicobacter pylori* infection is an important trigger for the development of precancerous lesions (Pellicano et al., 2016; Liou et al., 2019), additional risk factors include, diets high in sodium chloride (Tsugane et al., 2007), those rich in processed meat (Ward et al., 1997) ingestion of food containing carcinogens (Li et al., 1994), and diets poor in fruit and vegetables (Hertog et al., 1996), among others. More than 20 years ago it was hypothesized that a reduced functionality of the thyroid gland might also be involved (Venturi et al., 1993). Stomach and thyroid share the same embryological origin, and the epithelial cells from which the two organs originate are specialized in the uptake of iodine and its incorporation into various organic molecules (Venturi et al., 2000). For example, in humans, during a total‒body scintigraphy by using ^131^I, the radiotracer can be visualized in the stomach for more than 72 hours (Venturi et al., 1999). An association between iodine‒restricted goiter and GC was first reported about 90 years ago and confirmed by studies carried out in the 1950s (Spencer, 1954). In 1993, Venturi et al. advanced the hypothesis of a direct role of iodine deficiency on gastric tropism, since iodine competes for intracellular transport with various ions such as nitrates and thiocyanates in addition to chloride (Venturi et al., 1993). In a cohort of Chinese adults, including 29,584 subjects, in patients with goiter a statistically significant association with GC was found that persisted after adjusting for age, sex, tobacco smoking, body mass index (BMI), and even for a family history of GC (Abnet et al., 2006). Similarly, a higher prevalence of GC was observed in individuals in Italy from areas with a low‒iodine diet, such as farmers and populations living in mountains and hills, with respect to fishermen (Costa and Mortara, 1960). On the other hand, in human surveys iodine prophylaxis was shown to be effective in decreasing the GC incidence and death rate in iodine-deficient areas (Gołkowski et al., 2007). Goiter is in general considered a proxy of reduced thyroid function, given its association with both increased thyroid‒stimulating hormone levels (suggestive of clinical or subclinical primary hypothyroidism) (Dauksiene et al., 2017) and risk of autoimmune thyroid dysfunction (Aminorroaya et al., 2017), which are the leading causes of hypothyroidism in Western countries. Even though goiter prevalence partially decreased in Sardinia in the 1990s, due to dietary supplementation with iodized salt, and replacement therapy with L-thyroxine for hypothyroidism, its prevalence still remains around 16%-61% with large geographic variations (Loviselli et al., 2001). For example, in a recent study we outlined an area in *Ogliastra* as a pocket of endemic goiter (Tolu et al., 2019).

The purpose of ecological studies is to evaluate risk‒modifying factors on health based on populations defined either geographically or temporally. In this study, we aimed to detect a specific area in Sardinia with the highest rate of GC mortality and its relationship with hypothyroidism considered as the main predictor, using regression models taking into account spatial autocorrelation, while adjusting for potential confounders.

## Materials and Methods

*Setting*

Sardinia is the second-largest island in the Mediterranean Sea, located 120 kilometers west of the Italian coasts, with a total area of 24,100 square kilometers, and a population of 1,639,591 residents (Pes et al., 2017). The island encompasses 377 municipalities included into five provinces.

*Study design and measures*

As reported by Cossu et al., (2014) the mortality‒to‒incidence ratio for GC approximates the unity, thus enabling the use of mortality as a reliable proxy for incidence (Cossu et al., 2014). Raw data for GC mortality, recorded according to the International Classification of Diseases 9th revision (ICD‒9), were retrieved from the *“Atlante di Mortalità in Sardegna”* (Sardinia Mortality Registry) (Atlante della Mortalità Sarda 1981‒1988. ISTAT, Roma, Italy, 2005), which provided the number of observed and expected deaths for GC in each municipality, and allows the calculation of adjusted SMR. A ratio greater than 1.0 indicates that more than expected deaths have occurred and vice versa. Data on the prevalence of endemic goiter in the 377 Sardinian municipalities were collected from historical sources dating back to the first half of the 20^th ^century (Desogus, 1938; Ottonello, 1927; Putzu, 1928). At that time, before the advent of modern diagnostic testing, the vast majority of goiter cases were attributable to iodine deficiency and were mostly associated with an underactive thyroid gland. For this reason, in the present study goiter was considered as a proxy of reduced thyroid function in a sizeable proportion of the population. Additional variables used in the statistical analysis as potential confounders were: (i) a semi-quantitative score for the quality of diet, available for all villages (Fermi, 1934) and coded as an ordinal variable ranging from one (poorest diet) to five (richest diet); (ii) the average stature of the population, retrieved from the Fermi’s database (Fermi, 1934), since GC has been associated with short stature (Camargo et al., 2014), and the Sardinian population living in the mountain areas is well known to have the shortest stature in Europe (Pes et al., 2017); (iii) the prevalence of pastoralism (sheep rearing), considered as a proxy of *H. pylori* infection, given the very high prevalence of the infection in Sardinian shepherds, likely transmitted through sheep’s milk (Dore et al., 1999a; Dore et al., 1999b).

*Statistical analysis*

To search for clusters of GC mortality across the 377 island municipalities a multistep approach was adopted, running first a Kulldorff spatial scan (Kulldorff and Nagarwalla, 1995) taking each municipality as the smallest statistical unit by using the package ‘SpatialEpi’ implemented in the open‒source “R” software (http://www.r-project.org/). Briefly, the standardized mortality ratio for GC was calculated using the formula: SMR_i _= O_i_/E_i_ where O_i_ denotes the observed number of deaths and Ei the expected number of deaths in the i-th municipality (*i* = 1,…, 377). The SMR_i_ for GC, calculated in each municipality i, was taken as the outcome. Standardized mortality ratio for GC was tested for normal distribution with Kolmogorov‒Smirnov test and for homogeneity of variances with Levene’s test. Since it showed a nearly normal distribution, based on the results of the linearity tests, there was no need of transformation before analysis. Based on the reported number of deaths for GC in each municipality several circular areas were constructed as scanning windows, containing a pre‒set proportion of deaths, and a likelihood ratio test statistic with a significance level of 5% was run, assuming a Poisson probability distribution of the data. The area maximizing the test statistic, using the Monte Carlo simulation, was selected as a hotspot of GC mortality. This analysis was followed by spatial autocorrelation testing of all variables to detect a potential relationship between geographical location and the magnitude of goiter, prevalence adjusting for diet, stature and pastoralism. The global Moran’s I index (Moran, 1950) was calculated with the package ‘*ape*’ implemented in “R”. A significant positive or negative statistic indicates that locations of similar magnitude are spatially clustered or randomly dispersed, respectively. The next step was a local spatial regression model to analyze the association of GC mortality with potential predictors using a Geographically Weighted Regression (GWR) modelling. Geographically weighted regression 4.0 software for Windows with bisquare kernel function weighting and the adaptive bandwidth (Fotheringham et al., 2002), was used to generate parameter estimates for each geographical unit (Nakaya et al., 2005) according to the following formula:

${\mathrm{SMR}}_{i}=\sum_{k}\beta_{k}\left(u_{i},v_{i}\right)X_{k,i}+\in_{i}$

where SMR_i_ corresponds to the GC mortality ratio in the *i*-th municipality, coefficients β_k_ (u_i_,v_i_) are local parameters of the k-th independent variable and vary according to the location of each municipality, and ε_i_ is the Gaussian error at location *i*.

A map of regression coefficients for GC obtained from the GWR model was then generated to display the spatial distribution of parameters. The Aikake information criterion (AIC) was used as a measure the goodness of fit of the GWR model.

**Table 1:** Spatial Autocorrelation of study Variables

Variables	Global Moran’s I	p‒value
Gastric cancer SMR^1^	0.197	<0.0001
Goiter prevalence	0.118	<0.0001
Diet score	0.047	0.093
Body height	0.065	0.058
Pastoralism	0.111	<0.0001

^1^SMR, Standardized Mortality Ratio

**Figure 1:**
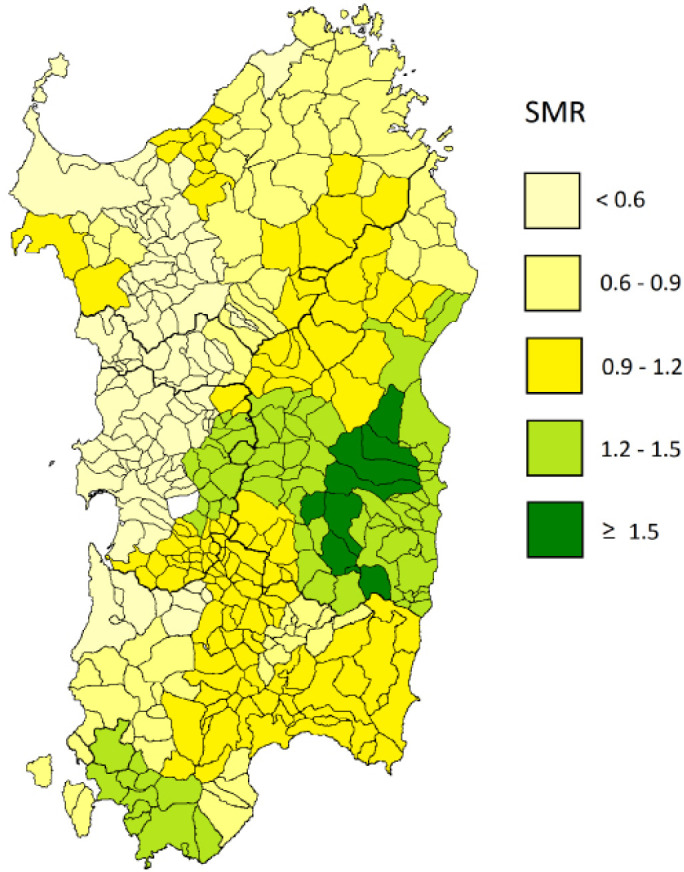
Standardized Mortality Ratio for Gastric Cancer in the 377 Municipalities of Sardinia

**Table 2:** Summary of the Local Geographically Weighted Regression Model

	Global regression		Geographically weighted regression				
	Estimate	SE	Min	25^th^ percentile	Median	75^th ^percentile	Max
Constant	0.2814	0.2068	-2.9731	-0.2402	0.3549	1.4054	4.2246
Goitre prevalence	0.2126	0.1222	-20.2134	-0.4630	0.1769	0.6547	9.4313
Diet score	-0.0318	0.0337	-1.0219	-0.2869	-0.0733	0.0538	0.6602
Body height	-0.0439	0.0554	-0.3432	-0.0760	-0.0428	0.0117	0.1932
Pastoralism	0.127	0.0805	-0.7553	-0.0576	-0.1503	0.2717	1.5133
Percent deviance							
explained	0.019				0.276		
AIC*	458.19				330.62		
Bandwidth size	50						

SE, standard error; AIC, Akaike information criterion

**Figure 2:**
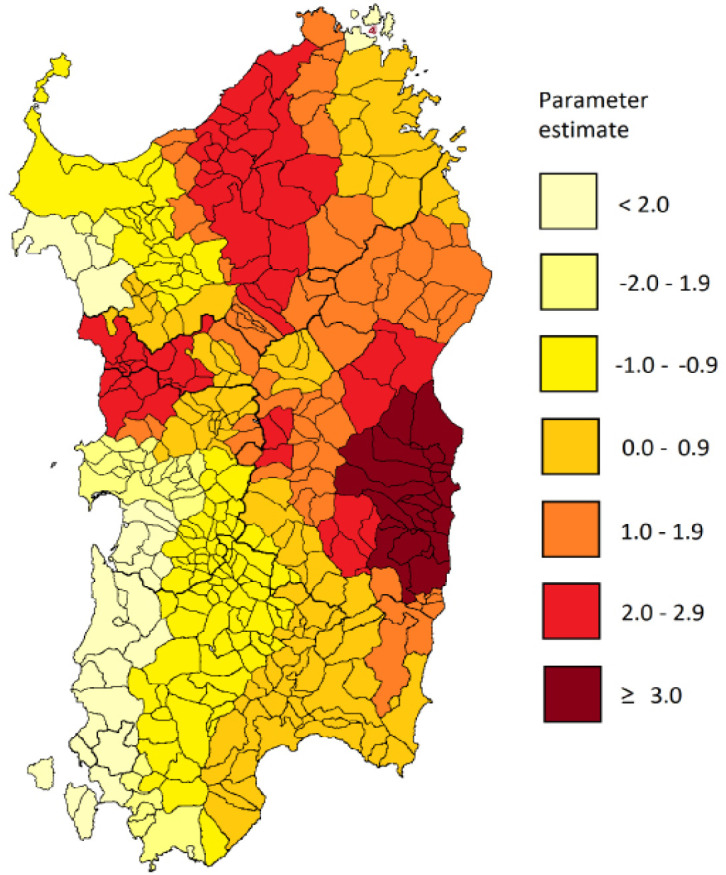
Spatial Distribution of the Local Coefficients for Goitre Prevalence in the Geographically Weighted Regression Model

## Results

*Detection of a spatial hotspot of GC mortality*

The SMR for GC in all municipalities ranged from 0.0 to 10.4 according to the geographical location. By using Kulldorff’s spatial scan statistics, a mortality hotspot was detected corresponding to the municipalities of *Talana, Urzulei, Villagrande, Arzana, Seui *and* Sadali *in the mountain our area, in part in the* Ogliastra *subregion, where GC mortality was nearly threefold higher than outside the hotspot (Figure 1).

*Spatial autocorrelation analysis*

Table 1 shows the global Moran’s *I* coefficient, an index of spatial autocorrelation, for all variables. Gastric cancer, goiter prevalence and pastoralism in all Sardinian municipalities displayed significant positive spatial autocorrelation, whereas diet score and average population stature did not show statistical significance.

*Geographically weighted regression model*

The GWR model was fitted including all study variables (Table 2). The model significantly improved the fitting compared with the global regression model (the AIC was reduced from 458.19 to 330.62 with a fixed bandwidth size of 50. Goiter prevalence was confirmed to be the predictor with the highest parameter value. Local diagnostic indicators (spatial coefficients for goiter prevalence) were mapped in Figure 2 and provided evidence that the model had a better explanatory power near the GC mortality hotspot. Spatial coefficients in males reached the highest values in the municipalities of Talana, Urzulei, Villagrande, Arzana, Seui and Sadali, indicating that the strength of the association was superior corresponding to the GC mortality hotspot. No significant associations were found in females (data not shown).

## Discussion

The spatial analysis conducted in this study identified a GC mortality hotspot in the *Ogliastra* mountain ous area, largely overlapping with an area of endemic goiter (Tolu et al., 2019). The GWR analysis confirmed a strong association between goiter prevalence and mortality for GC, consistent with the notion that GC is more frequent in areas where hypothyroidism is common (Venturi et al., 1993; Costa and Mortara, 1960; Gołkowski et al., 2007; Dore et al., 2020). For example, in Italian mountainous areas (i.e. the Tuscan‒Emilian Apennines) GC is more frequent and affects agricultural populations more than in fish‒eating ones (Costa and Mortara, 1960). In the specific case of the population living in *Ogliastra*, consumption of products of marine origin was very low before the advent of modern transportation and food preservation and storage (Pes et al., 2015). Until recently, the local diet consisted largely of dairy products (Pes et al., 2015) notoriously poor in iodine, and was reversed after supplementation of iodine in animal feeding (Nudda et al., 2009). Another possibility is that thyroid dysfunction could increase the risk of metabolic syndrome (Delitala et al., 2017; Delitala et al., 2019), which has been associated with GC (Li et al., 2018). Moreover, in a recent retrospective, case‒control, single‒center study, the prevalence of GC was significantly higher in males compared to females with hypothyroidism (1.4% versus 0.4%, p < 0.0001) (Dore et al., 2020). The increased risk in males with hypothyroidism (OR 5.10; p < 0.0001) remained after adjusting for potential confounders, especially for *H. pylori* infection.

Interestingly, in the present study the strong association of GC with goiter was significant only in males, in line with our recent clinical observation (Dore et al., 2020) reinforcing the hypothesis that the association is likely sex‒dependent and involves different mechanisms in men and women. The fact that the hotspot of GC mortality mainly affects men is intriguing and deserves further studies. At the moment we can speculate that women chronically affected by hypothyroidism are less likely to develop GC than men, irrespective of *H. pylori* infection and other environmental factors, due to the protective effect of estrogen (Iishi et al., 1993; Chandanos and Lagergren, 2008).

This study, being merely ecological, does not allow us to draw inferences about the underlying processes acting at the individual level. Speculative conjectures on the possible mechanisms behind this association might be put forward: (i) the presence of diffuse goiter in the population suggests persistent iodine deficiency. The thyroid gland is able to incorporate iodine in organic molecules with hormonal action, which physiologically control cell growth and differentiation (Venturi et al., 1993; Venturi et al., 2000). It is therefore reasonable to hypothesize that alterations of the thyroid gland (either in the direction of an increased or a decreased glandular function) can influence the risk of cancer proliferation. Consistent with this hypothesis, iodine prophylaxis was able to decrease the mortality for GC in iodine‒deficient areas (Gołkowski et al., 2007). Moreover, iodine deficiency might impair the immunological defense against tumor cells (Marani et al., 1985). Finally, among the molecular mechanisms that might mediate the action of thyroid hormones and gastric carcinogenesis it is interesting to note that in up to 50% of GC cases, a deletion in the gene coding for a subunit of nuclear receptor alpha for thyroid hormone THRA (OMIM 190120) was reported (Wang et al., 2002).

The present study also shows a statistically significant association of GC mortality with diet and pastoralism. It is known that the diet in *Ogliastra* was traditionally deficient in fruits and vegetables, favouring instead animal proteins (Pes et al., 2015). Although the traditional diet of the population of* Ogliastra* was considered particularly “healthy” (Buettner and Skemp, 2016) nonetheless some recent changes may be rather unhealthy. In general, it is known that processed meats including bacon, ham, beef jerky, corned beef, and other smoked or salted meat, among others were classified in 2015 as group 1 carcinogens, by the World Health Organization’s International Agency for Research on Cancer (Wiseman, 2008). In addition, there is a strong evidence, coming from several studies, that a high intake of salt‒preserved foods increases the risk of GC (Bouvard et al., 2015). Shikata et al., in a prospective study of patients classified according to salt intake, and followed for up to 14 years, observed that high dietary salt intake was a significant risk factor for GC. The risk was even stronger in patients with *H. pylori* infection and associated atrophic gastritis (Shikata et al., 2006). The population residing in* Ogliastra*, traditionally devoted to pastoral activities, had a relatively high consumption of meat, and in the absence of refrigeration, preservation was mainly carried out by the addition of salt (Cengarle et al., 2001). Several epidemiological studies reported a protective role of fruits (especially citrus fruit), and vegetables consumption against GC (Hertog et al., 1996), likely related to their vitamin C content, which can reduce the formation of N‒nitroso compounds in the stomach. A diet rich in fruits and vegetables reduces the GC risk by approximately 40% and 30%, respectively (La Vecchia et al., 1987; Riboli and Norat, 2003). In the* Ogliastra* region, and particularly in the municipalities encompassing the GC mortality hotspot, the consumption of fruit and vegetables was traditionally low (Pes et al., 2015) because the population, mainly devoted to sheep farming, did not cultivate fruit trees, favoring dairy products (Pes et al., 2013). According to this, the prevalence of pastoralism, and in turn of shepherds, was greater in the hotspot area. It has previously been reported that shepherds are frequently positive for *H. pylori* infection (e.g., 98%), and the sheep’s stomach may be a reservoir of the bacteria (Dore et al., 1999; Dore et al., 2001). However, in the present study, the strength of association with GC was statistically higher for goiter, similarly to our previous observation (Dore et al., 2020).

This study has several limitations. Being an ecological study, it implies the problem of ecological fallacy, and the impossibility of inferring a direct causality from mere association between aggregate data, although it may have an additional value for further exploratory research. Moreover, other risk factors for GC have not been considered such as smoking, alcohol consumption, and others. The number of variables available in all Sardinian municipalities for the period considered was limited, and unfortunately, we cannot rule out the existence of other potential confounders. 

In conclusion, goiter prevalence is as an independent predictor of GC mortality. Within the limitations of ecological studies, hypothyroidism seems to contribute to gastric carcinogenesis in males. Clinical research to better understand the relationship between gastric cancer and hypothyroidism is ongoing in this specific population to substantiate the possible effects of iodine on people’s stomach health. An increased understanding of factors influencing the GC risk would lead to more effective recommendations for surveillance in specific subgroups of patients.
